# Photothermally responsive theranostic nanocomposites for near‐infrared light triggered drug release and enhanced synergism of photothermo‐chemotherapy for gastric cancer

**DOI:** 10.1002/btm2.10368

**Published:** 2022-07-12

**Authors:** Taicheng Zhou, Lili Wu, Ning Ma, Fuxin Tang, Jialin Chen, Zhipeng Jiang, Yingru Li, Tao Ma, Na Yang, Zhen Zong

**Affiliations:** ^1^ Department of Gastroenterological Surgery and Hernia Center The Sixth Affiliated Hospital, Sun Yat‐sen University Guangzhou Guangdong China; ^2^ Guangdong Provincial Key Laboratory of Colorectal and Pelvic Floor Diseases The Sixth Affiliated Hospital, Sun Yat‐sen University Guangzhou Guangdong China; ^3^ Department of Medical Ultrasonics Third Affiliated Hospital of Sun Yat‐sen University, Guangdong Key Laboratory of Liver Disease Research Guangzhou Guangdong China; ^4^ Department of Clinical Laboratory Guangzhou First People's Hospital, School of Medicine, South China University of Technology Guangzhou Guangdong China; ^5^ Department of Gastroenterological Surgery The Second Affiliated Hospital of Nanchang University Nanchang Jiangxi China

**Keywords:** chemotherapy, gastric cancer, nanocarriers, photothermal therapy, photothermally responsive

## Abstract

Near‐infrared (NIR) photothermal therapy plays a critical role in the cancer treatment and diagnosis as a promising carcinoma treatment modalities nowadays. However, development of clinical application has been greatly limited due to the inefficient drug release and low tumor accumulation. Herein, we designed a NIR‐light triggered indocyanine green (ICG)‐based PCL core/P(MEO_2_MA‐*b*‐HMAM) shell nanocomposites (PPH@ICG) and evaluated their therapeutic effects in vitro and in vivo. The anticancer drug 5‐fluorouracil (5Fu) and the photothermal agent ICG were loaded into a thermo‐sensitive micelle (PPH@5Fu@ICG) by self‐assembly. The nanoparticles formed were characterized using transmission electron microscopy, dynamic light scattering, and fluorescence spectra. The thermo‐sensitive copolymer (PPH@5Fu@ICG) showed a great temperature‐controlled drug release response with lower critical solution temperature. In vitro cellular uptake and TEM imaging proved that PPH@5Fu@ICG nanoparticles can home into the lysosomal compartments under NIR. Moreover, in gastric tumor‐bearing nude mice, PPH@5Fu@ICG + NIR group exhibited excellent improvement in antitumor efficacy based on the NIR‐triggered thermo‐chemotherapy synergy, both in vitro and in vivo. In summary, the proposed strategy of synergistic photo‐hyperthermia chemotherapy effectively reduced the 5Fu dose, toxic or side effect, which could serve as a secure and efficient approach for cancer theranostics.

## 1 INTRODUCTION

As one of the most common malignant cancer types of the digestive system, gastric cancer (GC) is the second leading cause of cancer‐related deaths worldwide.^1^, ^2^, ^3^ Current therapies for GC, such as surgery, chemotherapy, and radiation therapy, are not yet satisfactory.^4^ Thus, there is an urgent need for developing new therapeutic approaches for the treatment of GC. Polymeric nano drug carrier for GC chemotherapy plays a vital role in promoting the drug bioactivity and biocompatibility during the past decades, and have achieved certain therapeutic effects.^5^ Recently, light triggered therapeutic approaches like photothermal therapy (PTT) for cancer treatment has received considerable attention due to its numerous superiority, such as minimal invasion, high spatiotemporal precision, and localized treatment.^6^, ^7^, ^8^ It has been reported that near‐infrared (NIR) photothermal agents, such as fluorescent dyes, gold nanomaterials, carbon nanotubes, and graphene oxide, could strongly absorb NIR laser and trigger temperature increase to induce the rapid drug release in thermo‐responsive system.^9^, ^10^ The photothermo‐chemotherapy employed NIR photosensitizer to generate hyperthermia (above 43°C) in target tumor regions by remotely controlled NIR‐irradiation, which leaded to synergism by thermal ablation of cancer cells and enhanced antitumor drug release with significantly reduced toxic side effects, which are commonly produced by the single strategies like chemotherapy, radiotherapy, and surgery.^11^, ^12^

Indocyanine green (ICG) has been widely applied to biomedical imaging diagnosis and PTT with NIR fluorescence and 808 nm spectral absorption peak.^13^, ^14^ ICG has been approved for human use by Food and Drug Administration as a diagnostic cyanine NIR dye, which has ability of converting NIR light into heat.^15^, ^16^ Currently, NIR laser triggered hyperthermia has been developed to remotely control drug release in nanoparticles. NIR laser is an optimal choice for in vivo cancer therapy due to weaker tissue bioluminescence in 650–900 nm regions and less interference of biological background to fluorescence signal, which could provide strong tissue penetration for deep tissue imaging.^17^, ^18^, ^19^ Controlled release of anticancer drugs could be released through NIR laser‐triggered hyperthermia of thermo‐responsive nano‐carriers.^20^ Notably, due to the different degree of the temperature sensitivity, hyperthermia can induce the death of cancer cells but not normal cells.^20^ However, the applications of free ICG in NIR fluorescence imaging and PTT faced a series of restrictions owing to its drawbacks, including instability in aqueous solution and quick body clearance.^21^, ^22^

To overcome these challenges, various strategies have been developed to encapsulate ICG into nanocarriers, which provide enhanced prolonged circulation times and fluorescence stability in vivo.^23^, ^24^ The hyperthermia from ICG‐micelles can lead to phase transition from the gel phase to the liquid crystalline phase, which promotes the drug release from micelles. Therefore, the ICG‐loaded micelles would provide a new platform for cancer therapy to achieve a combinative effect of chemotherapy and photothermotherapy.^25^ To achieve the NIR‐triggered drug release and enhanced synergism of photothermo‐chemotherapy, various stimuli‐responsive nanocarriers respond to external stimulations (light, hyperthermia, magnetic field, and ultrasound) and internal stimulation (reduction/oxidation, pH, and enzyme) have been explored with perfect performances.^26^, ^27^ Thermo‐responsive micelles have been deeply researched in oncotherapy and exhibit an enormous advantage in intelligent drug release among all the stimuli‐responsive nanoparticles.^28^ The thermo‐responsive micelles realize the controlled drug release by a gel‐to‐liquid phase transition of thermo‐responsive polymers in the region of their phase transition temperature, and the permeability of nanoparticles increases due to the instability of the shell, which facilitate rapid drug release.^29^, ^30^ Delivery efficiency of chemotherapeutic agent can be enhanced through optimizing the lower critical solution temperature (LCST) of the thermo‐sensitive polymer and hyperthermia temperature of ICG.

**SCHEME 1:**
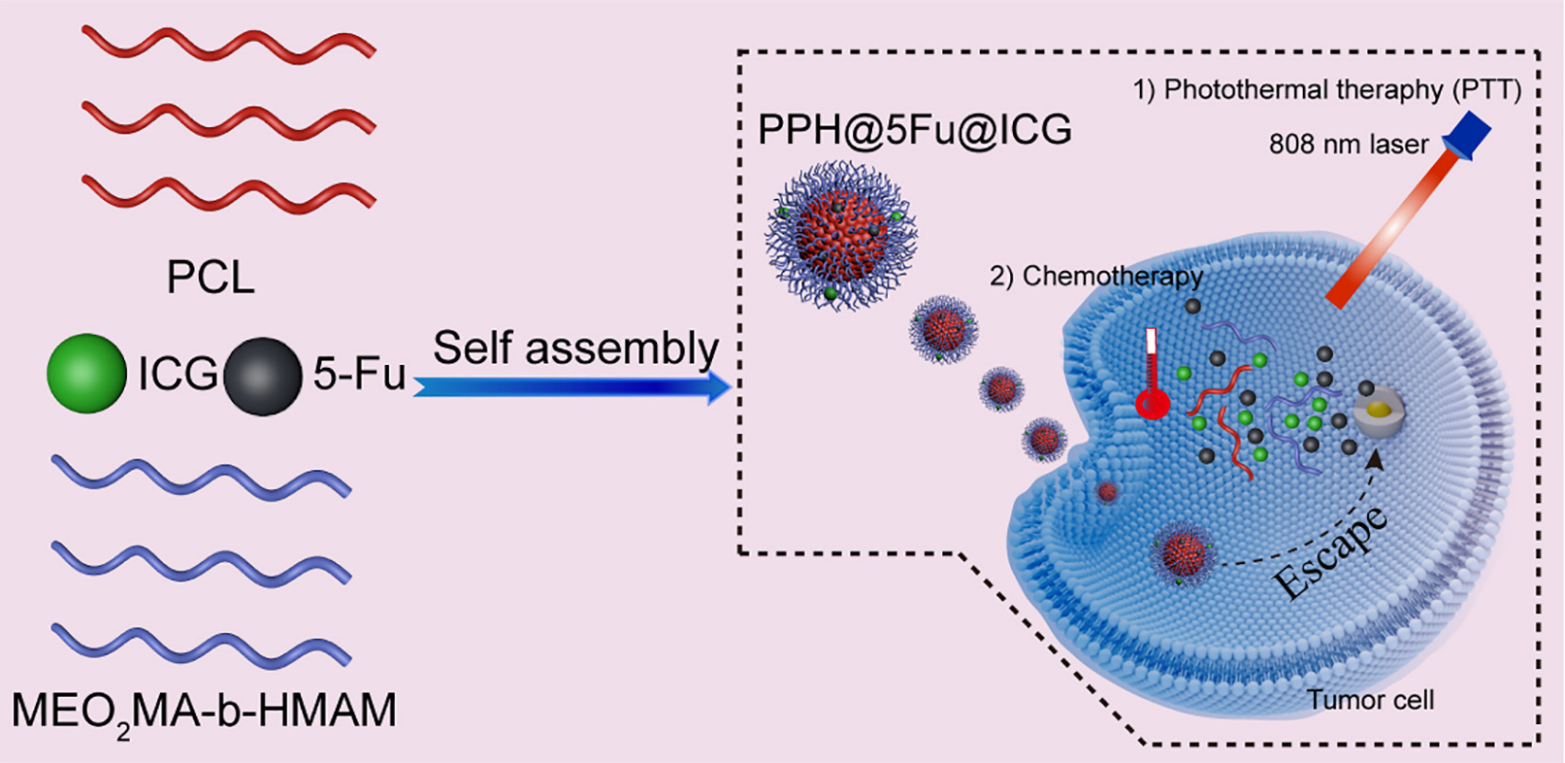
Schematic illustration for the preparation of PPH@5Fu@ICG and its related mechanisms for the treatment of GC

Designing a new nano‐platform that integrates a variety of imaging and treatment components for treatment of cancer therefore remains a challenge. In this study, 5‐fluorouracil (5Fu) and ICG co‐encapsulated thermo‐sensitive micelles (PPH@5Fu@ICG) was prepared by self‐assembly method (Scheme 1). This unique design could inhibit tumor growth through real‐time tracking, NIR laser‐driven drug release, and chemo/PTT. 5Fu, an agent for chemotherapy, is used as one of the standard chemotherapy regimens for GC.^31^, ^32^ The physiochemical characters were systematically investigated to explore the mechanism for drug release of PPH@5Fu@ICG. Fluorescence (FL) of ICG in PPH@5Fu@ICG was monitored to demonstrate subcellular localization, NIR laser‐driven drug release, and metabolic distribution. The cytotoxic effects of PPH@5Fu@ICG combined controllable drug release, chemotherapy, and PTT were gradually evaluated in GC cells. Finally, the antitumor efficacy of PPH@5Fu@ICG in vivo through intratumoural injection was further appraised in comparison with single strategy. Strikingly, the design is expected to use thermo‐sensitive micelles to overcome the immune escape induced by chemotherapy, and reduce the side effects of chemotherapy through the photo‐thermal response of ICG. The outcome of our work may be considered as a promising approach to integrate chemotherapy and PTT for GC therapy.

## 2 EXPERIMENTAL SECTION

### 2.1 Materials and reagents

The reagents ICG (purity ≥98%), N‐hydroxymethyl acrylamide (HMAM, analytically pure), trimethylamine, 2‐(2‐methoxyethoxy)ethyl methacrylate (MEO_2_MA, analytically pure), and 2‐bromoisobutyryl bromide (analytically pure) were obtained from Sigma Aldrich Corporation (MO, USA). 5‐Fluorouracilorubicin (5Fu, purity ≥99%) was purchased from Macklin Biochemical Co., Ltd (Shanghai, China). Ɛ‐Caprolactone (CL, 99%, Sigma) was purified with CaH_2_ for 48 h at room temperature and distilled under reduced pressure before use. Sn(Oct)_2_ (analytically pure) was distilled under reduced pressure before use. MEO_2_MA was passed through a column of activated basic alumina to remove inhibitors. Dipentaerythritol was dried in vacuo at 60°C for 24 h before use. Copper(I) bromide (CuBr, Aldrich, 98%) was purified via stirring in acetic acid and washing with ethanol and then dried in a vacuum. 2‐Bromoisobutyryl bromide was used without further purification.

Calcein AM, Alexa Fluor 488 Annexin V/Propidium Iodide (PI) Cell Apoptosis Kit, and cell counting kit‐8 (CCK‐8) were obtained from KeyGen Biotech Co., Ltd (Nanjing, China). Calcein‐AM and one Step TUNEL apoptosis Assay Kit were purchased from Invitrogen (Oregon, USA). Fetal bovine serum, RMPI 1640, high‐glucose Dulbecco's modified Eagle's medium (DMEM), trypsin–EDTA and penicillin–streptomycin were purchased from Gibco (Carlsbad, USA).

### 2.2 Polymer synthesis

#### 2.2.1 Synthesis of poly(ε‐caprolactone)

Poly(ε‐caprolactone) (PCL) was synthesized by ring‐opening polymerization (ROP) of CL using dipentaerythritol as an initiator and Sn(Oct)_2_ as a catalyst.^33^ Briefly, CL (20.54 g, 180 mmol), dipentaerythritol (0.30 g, 1.18 mmol), and a catalytic amount of Sn(Oct)_2_ were added to a flame‐dried polymerization tube. The reaction tube was then flushed with argon and evacuated three times. After the stirring of the mixtures with a magnetic stirrer for 24 h at 115°C, the obtained crude product was dissolved in dichloromethane and then precipitated with iced methanol three times. Finally, the above samples were dried in a vacuum oven at 40°C to constant weight.

#### 2.2.2 Synthesis of PCL‐Br

The PCL‐Br was synthesized through esterification of 2‐bromoisobutyryl bromide (BIBB), and the specific operations were as follows: 10 g of PCL (containing 3.46 mmol hydroxyl) was dissolved in 50 ml of dried chloroform under the protection of nitrogen atmosphere, and 1.5 g of triethylamine (14.82 mmol) was added. Then, the solution was cooled to 0°C in an ice bath, and BIBB (2.382 g, 10.36 mmol) dissolved in 20 ml of dried chloroform was added to the above system. The entire reaction system was stirred at room temperature for 24 h under argon protection, then filtered to remove insoluble salts. The filtrate was quenched three times with chloroform and deionized water (1:1, v/v). The organic phase was collected, and then solvents were removed by using a rotary evaporator. Solid products were precipitated 2 times in iced methanol and dried in vacuum at room temperature to constant weight.

#### 2.2.3 Synthesis of PCL‐*b*‐(MEO_2_MA‐*co*‐HMAM)

The PCL‐*b*‐(MEO_2_MA‐*co*‐HMAM) block copolymers were synthesized by atom transfer radical polymerization (ATRP) using a “grafting from” strategy.^34^ Typical procedure was outlined as follows: CuBr (28.7 mg, 0.20 mmol), PCL‐Br macroinitiator (1.0 g, containing 0.33 mmol of C‐Br groups), MEO_2_MA (3.117 g, 16.56 mmol), OEGMA (0.684 g, 1.44 mmol), and THF (15 ml) were sequentially added into the oven‐dried reaction flask and stirred with a magnetic stirring bar. The air of the reaction flask were degassed for three times by freeze‐pump‐thaw cycles. Next, PMDETA (34.9 mg, 0.20 mmol) was added into the reaction flask by using a syringe under an argon atmosphere. After reaction at 55°C for 5 h, the resulting product was dissolved in THF and passed through a basic alumina column to remove the copper catalysts. The obtained polymer was precipitated into ice‐cold hexane and dried in vacuum at room temperature to constant weight. The chemical structure of PCL‐*b*‐(MEO_2_MA‐*co*‐HMAM) was analyzed by nuclear magnetic resonance (^1^H NMR) spectrometer (AVANCE III 500M, Bruker, Germany).

### 2.3 Formulation and characterization of PPH@5Fu@ICG

The ICG‐loaded micelles were prepared as follows: PPH@5Fu@ICG were synthesized from PCL‐*b*‐(MEO_2_MA‐*co*‐HMAM), 5Fu, and ICG using self‐assembly method. First, PCL was dissolved in THF solution at a concentration of 1 mg/ml. Second, 0.5 mg of PCL‐*b*‐(MEO_2_MA‐*co*‐HMAM), 450 μg of 5‐fluorouracil, and 450 μg of ICG were added in 5 ml THF, and solution was added dropwise under sonication using an ultrasonics processor (FS‐450, Branson, USA) at a power of 200 W for 5 min. Afterward, blank polymer micelles were prepared by dissolving the PCL‐*b*‐(MEO_2_MA‐*co*‐HMAM) copolymer in THF and then added dropwise into 10 ml distilled water within 30 min under magnetic stirring. Next, the mixture was dialyzed against ultrapure water by using a dialysis membrane (8000–14,000 Da molecular weight cut‐off) for 36 h. The ultrapure water was refreshed every 6 h to remove the organic solvent. Finally, the micellar solution was diluted into different concentrations with deionized water or a phosphate buffer solution (PBS; pH 7.4) for the following experiments. The same procedures were used to prepare the ICG‐loaded micelles (PPH@ICG) and 5‐fluorouracil‐loaded micelles (PPH@5Fu). All samples were filtered by 0.45 μm filter membrane in next experiments. TEM images of micelles with or without 5Fu encapsulating were further observed by TEM (JEM‐100XII, Tokyo, Japan) at 37°C and 43°C. The size distribution and surface charge of the micelles at 37°C and 43°C were determined on a Zetasizer analyzer (Zetasizer Nano ZS90, Malvern, UK). The fluorescence intensity of ICG and PPH@ICG was measured by using a spectrofluorometer (F‐2500; Hitachi, Japan). The transmittance of PPH@5Fu@ICG with temperature variation was measured by UV spectrophotometer (UNICO UV‐1800, Shanghai, China) at 600 nm wavelength. DSC experiments was measured by a DSC instrument (TA Q2000, New Castle, DE) at a scan rate of 1°C/min.

### 2.4 NIR laser‐triggered photothermal response in vitro

ICG (40, 60, and 80 μg/ml) and PPH@ICG (ICG: 40, 60, and 80 μg/ml) were dissolved in PBS and added into 6‐well plate. The samples was irradiated by 808 nm NIR laser (1 W/cm^2^, 5 min). The PPH@ICG solution (ICG: 40 μg/ml) was irradiated by 808 nm NIR laser for 1 min, and then the irradiation was stopped to lower the temperature to the initial temperature. The experimental operation was carried out repeatedly for six times. The thermocouple thermometer is inserted into the liquid surface at room temperature and the temperature is recorded every 20 s. Thermographic camera images and temperatures were obtained by infrared imaging camera (Ti27, Fluke, USA).

### 2.5 NIR triggered drug release in vitro

The encapsulation efficiency (EE) and drug loading capacity (LD) of PPH@5Fu@ICG were measured by a UV–vis spectrometer at 265 nm. The standard absorption curve of 5Fu in PBS was obtained and the formulas used were as follows:

EE (%) = (weight of 5Fu in micelles)/(weight of feed 5Fu) × 100%.

LD (%) = (weight of 5Fu in micelles)/(weight of micelles) × 100%.

A dynamic dialysis method was used to determine the release rates of NIR‐triggered release of PPH@5Fu@ICG in vitro. Briefly, 3 ml PPH@5Fu@ICG aqueous solution was added into dialysis bags (MW, 3500 Da) and immersed into 200 ml PBS (pH 7.4) at 37°C, with 808 nm laser irradiation (1 W/cm^2^, 5 min) and 45°C for 24 h, respectively. At each preset‐time point, 1 mL dialysate was collected and the same volume of fresh PBS was added. The cumulative release curve of PPH@5Fu@ICG were measured by a UV–vis spectrometer.

### 2.6 Cell culture experiments

For in vitro experiments, human GC AGS cells were obtained from Chinese Academy of Sciences (Shanghai, China) and were kept at 37°C in a 5% CO_2_ humidifier incubator. The DMEM media supplemented with 10% FBS, 1% penicillin, and 1% streptomycin were used as the culture media.

### 2.7 Cellular uptake

AGS cells were seeded onto cover‐slide system at a density of 5 × 10^4^/well and cultured overnight for cell attachment. Then the cells were treated with PPH@5Fu@ICG (containing 40.0 μg/ml ICG) without or with 808 nm laser irradiation (1 W/cm^2^, 5 min) after 4 h and 12 h incubation. The cells were washed for three times with PBS, fixed with cold 4% paraformaldehyde for 2 h, and stained with 1 μg/ml DAPI for 15 min. Finally, the cellular internalization were observed by a confocal laser scanning microscope (LSM710, Leica, Germany).

For further quantification, AGS cells were seeded onto 12‐well plates with a density of 1 × 10^5^/well and cultured overnight for cell attachment at 37°C. Then medium was replaced and cells were incubated with blank medium (Control), PPH@5Fu@ICG (containing 40.0 μg/ml ICG) without or with 808 nm laser irradiation (1 W/cm^2^, 5 min), respectively. The cells were re‐suspended in PBS and quantitatively evaluated by flow cytometry (FACSAriaII, BD, USA). Additionally, cells were collected and fixed in 2.5% glutaraldehyde, and the morphology of cells were observed by TEM to examine changes in the ultrastructure of the cells.

### 2.8 Cellular cytotoxicity analysis

The antitumor activities of the micelles were evaluated on AGS cells using CCK‐8 assays. Cells were seeded at a density of 5 × 10^4^/ml in a 96‐well plate for incubation. After overnight culturing, AGS cells were treated by PPH or PPH@5Fu at different concentrations. After 24 h further incubation, medium was removed and 20 μl CCK‐8 solutions (5 mg/ml) were added. Cells were then incubated for 4 h at 37°C, the cytotoxicity of PPH or PPH@5Fu was analyzed by the CCK‐8 assay. Likewise, we analyzed the cyotoxicity of PBS + NIR, PPH@5Fu, PPH@5Fu@ICG, PPH@ICG + NIR, and PPH@5Fu@ICG + NIR by the same assay.

Flow cytometry was further used to evaluate the cytotoxicity of PBS + NIR, PPH@5Fu@ICG, PPH@ICG + NIR, and PPH@5Fu@ICG + NIR. AGS cells were seeded onto 24‐well plates at a density of 5 × 10^4^/well and cultured overnight. Cells were then treated with PBS + NIR, PPH@5Fu@ICG, PPH@ICG + NIR, and PPH@5Fu@ICG + NIR at different concentrations, respectively. After 24 h incubation, AGS cells were harvested. Cell apoptosis was detected with Alexa Fluor 488 Annexin V/PI Cell Apoptosis Kit for flow cytometry (FACSAriaII, BD). Herein, Annexin V‐positive and PI‐negative cells were scored as apoptotic, and both Annexin V‐positive and PI‐positive cells were scored as late apoptotic/necrotic cells.

### 2.9 In vivo tumor model

All animal protocols were reviewed and approved by the Institutional Animal Care and Use Committee (IACUC) of the Sixth Hospital of Sun Yat‐sen University. Female BALB/c nude mice (6 weeks old, 19–23 g) were purchased from Nanfang Hospital, Southern Medical University (Guangdong, China). Mice were settled in cages in groups (five mice per cage), and fed with standard water and mouse chow. To establish tumor model, 4 × 10^6^ AGS cells were planted by subcutaneous injection into the right leg areas of male nude mice. Tumor size was measured three times a week and the tumor volume was calculated (*V* = (tumor length) × (tumor width)^2^/2). When the tumor volume of mice reached 100–200 mm^3^, nude mice bearing AGS tumor were randomly divided into seven groups (*n* = 3), followed by injection with 100 μl of PBS, PBS + NIR (808 nm, 1 W/cm^2^, 5 min), PPH, PPH@ICG + NIR (808 nm, 1 W/cm^2^, 5 min), 5Fu, PPH@5Fu@ICG, and PPH@5Fu@ICG + NIR (808 nm, 1 W/cm^2^, 5 min), respectively. The tumor volumes and changes in body weight of mice of each group were monitored once every 2 days for a total of 14 days. After the last measurement, mice were sacrificed by cervical dislocation under an anesthetic status and the major organs of mice were collected for H&E staining. Moreover, immunofluorescence (IF) staining of CD31, Ki67 and TUNEL were performed on tumor slices to assess the antitumor efficacy of each experimental group according to the manufacturers' instructions.

### 2.10 In vivo fluorescence imaging and biodistribution analysis

At indicated time points (2, 4, and 12 h), FL images of ICG and PPH@ICG were acquired after injection utilizing an in vivo imaging system (Cri Maestro, PerkinElmer). The signal of ICG was collected with a 730 nm excitation wavelength and a 750 nm filter. The temperature change of mice tumor were obtained by infrared imaging camera. The mice after 12 h injection were sacrificed and the major organs (heart, liver, spleen, lung, and kidneys) were collected for in vivo imaging and biodistribution analysis.

### 2.11 Statistical analysis

All the quantitative results were presented as mean ± standard deviation. Students's *t*‐test was utilized to evaluate the significance in two groups, and one‐way ANOVA was used among multiple groups. Herein, a probability value *P* < 0.05 (*) was accepted as statistically significant difference, and double asterisk (**) indicated *P* < 0.01, was considered as very significant difference.

## 3 RESULTS AND DISCUSSION

### 3.1 Synthesis and characterization of the temperature‐sensitive PPH

PCL‐*b*‐(MEO_2_MA‐*co*‐HMAM) copolymers were obtained by the combination of living ROP and ATRP. ^1^H NMR characterization of the PCL‐Br and PCL‐*b*‐(MEO_2_MA‐*co*‐HMAM) is presented in Figure S1A. The peak of methylene protons is detected at *δ* 4.06 ppm, as well as the other peaks (1.38, 1.65, and 2.31 ppm) were ascribed to the protons of PCL. Terminal methylene protons (d’) are found at *δ* 3.65 ppm. PCL‐Br macroinitiators were prepared by coupling excess 2‐bromoisobutyryl bromide with the terminal hydroxyl groups of PCL. The molecular weight of PCL‐Br increased slightly and the molecular weight distribution was similar to that of PCL. The ^1^H NMR spectrum of PCL‐*b*‐(MEO_2_MA‐*co*‐HMAM) indicated a new peak at *δ* 1.93 ppm (methyl protons of 2‐bromoisobutyryl bromide) and the complete disappearance of the peak at *δ* 3.65 ppm. These results indicated complete reactions of all terminal hydroxyl groups of PCL. PCL‐*b*‐(MEO_2_MA‐*co*‐HMAM) was prepared by ATRP of MEO_2_MA and OEGMA co‐monomers.

FTIR spectrometry was utilized to further confirm the synthesis of polymer. As shown in Figure S1B, the peak at 3680 cm^−1^ and 1740 cm^−1^ was the absorption band of the hydroxyl group and ester bond of PCL segments. The C–H symmetric stretching vibration peak of CH_3_ and CH_2_ was observed at 2950 cm^−1^ and 1730 cm^−1^. Additionally, the C—O—C stretching vibration band at 1180 cm^−1^ and the C=O stretching band at 1750 cm^−1^ were observed both in PCL‐Br. The stretching vibration peak of C—O—C at 1178 cm^−1^ was observed in PCL‐*b*‐(MEO_2_MA‐*co*‐HMAM) and relative intensities of the absorptions are stronger than those of PCL‐Br. Moreover, new absorption peak at 3680 cm^−1^ and 3300 cm^−1^ appear in PCL‐*b*‐(MEO_2_MA‐*co*‐HMAM), which indicated the presence of the —OH bonds and —NH bonds. By GPC analysis, the weight‐average molecular weights (Mw) of PCL, PCL‐Br, and PCL‐*b*‐(MEO_2_MA‐*co*‐HMAM) was 15,850, 16,750, and 59,370 Da, respectively (Figure S2).

**FIGURE 1:**
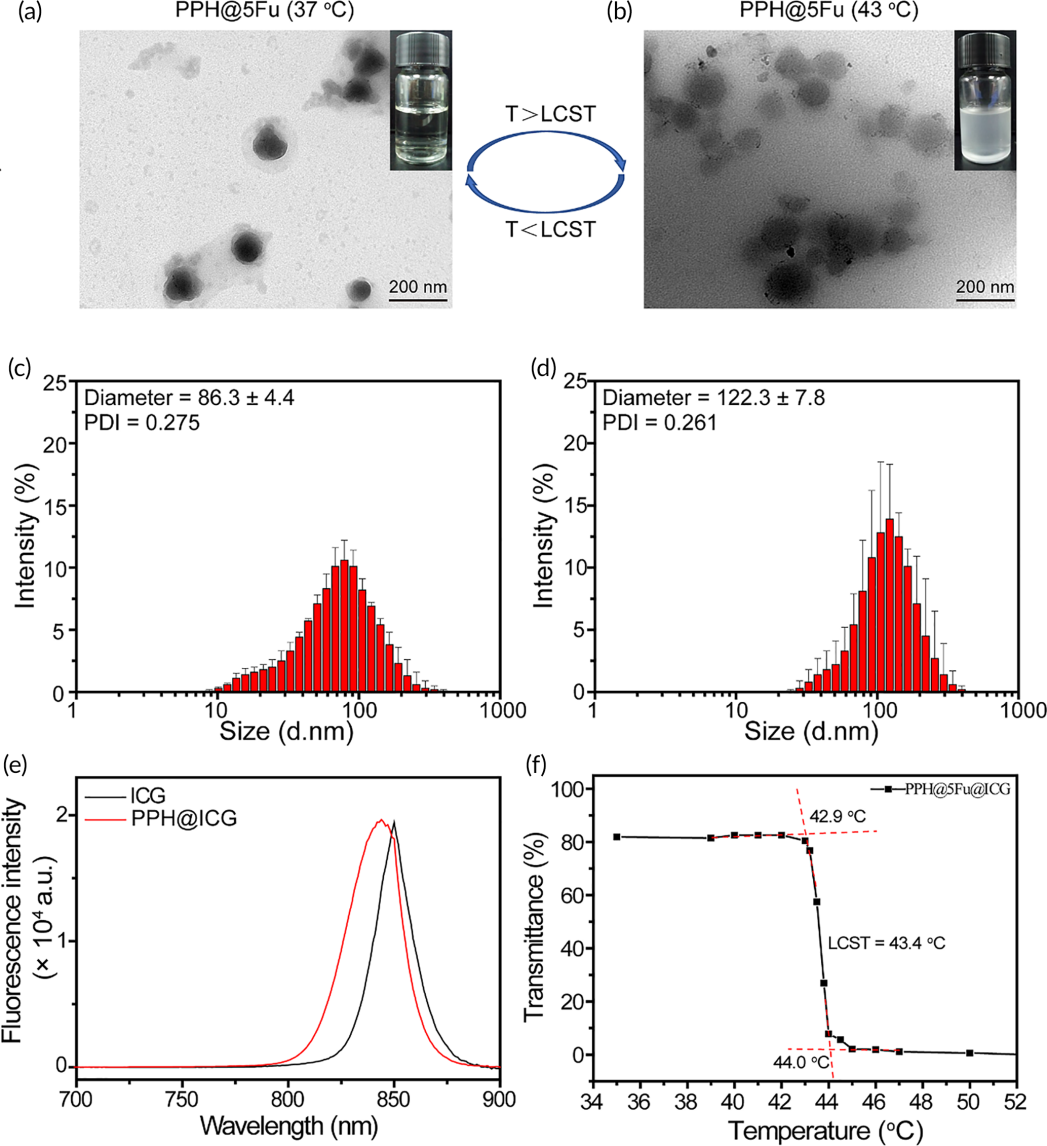
Characterization of the temperature‐sensitive PPH@5Fu nanoparticles. (a) TEM images of PPH@5Fu nanoparticles at 37°C. (b) TEM images of PPH@5Fu nanoparticles at 43°C. (c) Size distribution of PPH@5Fu nanoparticles at 37°C. (d) Size distribution of PPH@5Fu nanoparticles at 43°C. (e) The fluorescence emission spectra of ICG and PPH@ICG in the wavelength from 700 to 900 nm. (f) The transmittance curves of PPH@5Fu@ICG micelles

Blank polymer micelle (labeled PPH) is composed of PCL, MEO_2_MA, and HMAM, which was utilized to co‐encapsulate hydrophobic anticancer drug 5Fu and theranostic photo‐thermo agent (ICG). Temperature‐responsive PPH@5Fu was assembled using PCL, MEO_2_MA, HMAM, and 5Fu. PPH can undergo an irreversible phase transition to realize rapid release of encapsulated drugs as the temperature rises higher than 43°C. As shown in Figure S3A,B, the morphology of PPH at 37°C and 43°C exhibited excellent monodispersity, generally spherical shape and uniform size distribution. The hydrodynamic size of PPH at 37°C and 43°C was 60.7 ± 4.6 nm and 112.8 ± 5.8 nm, respectively (Figure S3C,D). As shown in Figure 1a,b, the morphology of PPH@5Fu at 37°C and 43°C exhibited larger spherical shape than PPH. The hydrodynamic size of PPH@5Fu at 37°C and 43°C was 86.3 ± 4.4 nm and 122.3 ± 7.8 nm, respectively (Figure 1c,d). The increase of the particle size in PPH@5Fu at 43°C was may due to that the loaded drug will fill the cavity. Prior study revealed that nanoparticles with small size were much easier to be internalized by tumor cells and could penetrate into deeper areas of tumors for cancer therapy.^35^ Compared with commercial liposome (liposome doxorubicin ~220 nm), PPH@5Fu with smaller size (86.3 nm) showed great potential to penetrate into deep tumors for efficient treatment. The absorption spectra of free ICG and PPH@ICG were measured by UV–Vis spectrophotometry. As shown in Figure 1e, PPH@ICG showed a strong NIR absorption of at around 783 nm, showing a blue shift of 18 nm compared with the 801 nm absorption peak of free ICG, which indicated that PPH@ICG fully met the requirements of PTT using an 808 nm laser. The critical micelle concentration was 45.4 mg/ml. The blue shift may be due to the surface effect. After encapsulating the ICG with nanomaterials, a composite nanomaterial is formed, resulting in smaller particles and larger specific surface area, which further resulted in lattice distortion, smaller lattice constants, and shortened bond lengths. As shown in Figure 1f, LCST was 43.4°C. As shown in Figure S4, DSC curves of PPH@5Fu@ICG demonstrated LCST, indicating general agreement of both results.

**FIGURE 2:**
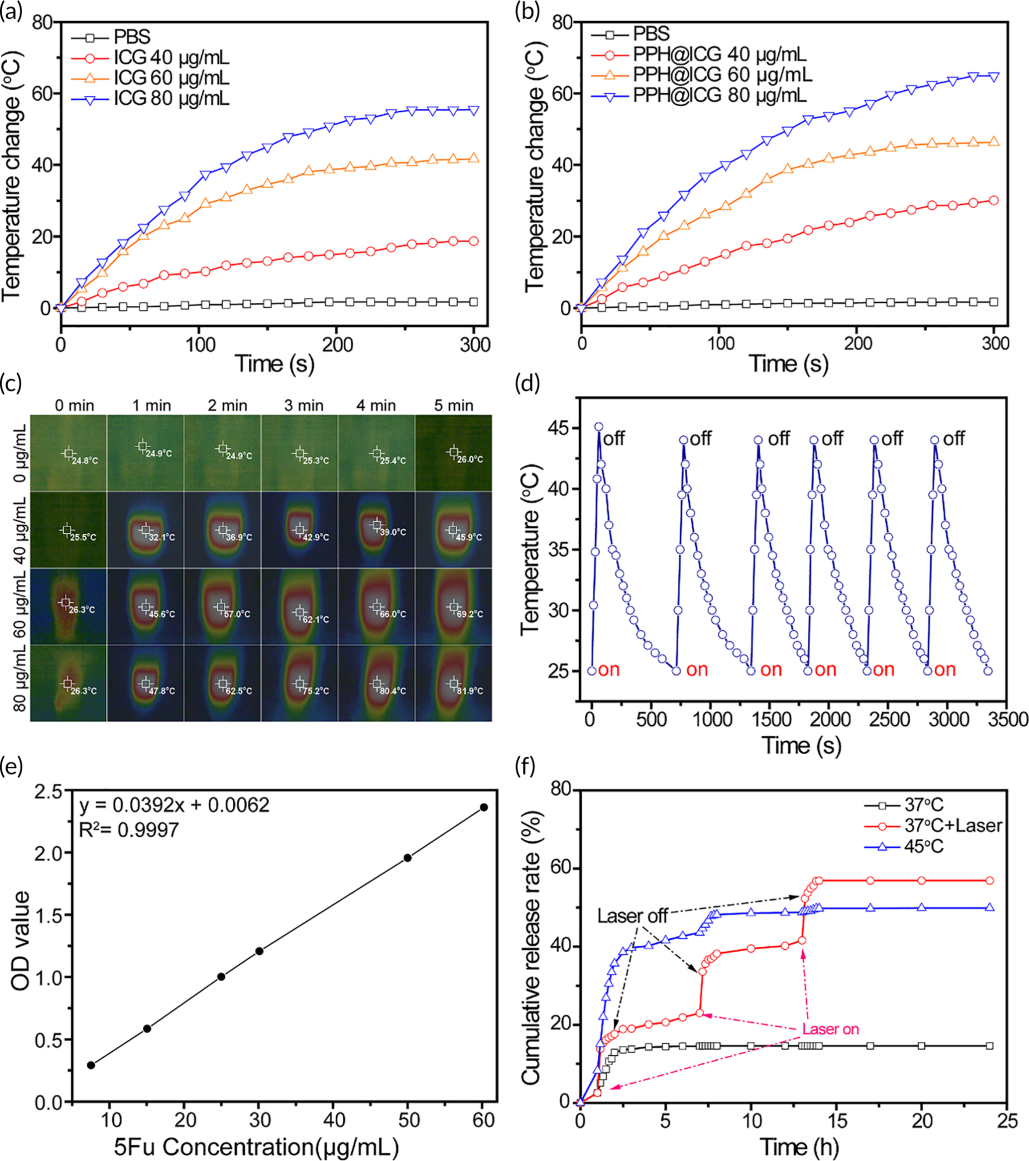
Photothermal properties of the nanoparticles. Photothermal heating curves of free ICG (a) and PPH@ICG (b) with various concentrations under continuous laser irradiation (808 nm, 1 W/cm^2^). (c) Infrared thermographs of PPH@ICG under continuous laser irradiation (808 nm, 1 W/cm^2^) for various irradiation times. (d) Photothermal stability evaluation of PPH@ICG with laser switch‐on and switch‐off treatment for six cycles. (e) The standard curve of 5Fu. (f) In vitro cumulative 5Fu release rate of PPH@ICG@5Fu at 37°C (with or without of laser irradiation) and 45°C. 5Fu, 5‐fluorouracil; ICG, indocyanine green

ICG is a member of cyanine that generally consists of two heterocyclic units connected with linear alkene units. When irradiated by NIR laser, ICG molecules absorb luminous energy and reach to the high vibration energy level of excited state. Then, ICG molecules generate thermal energy and act out by hyperthermia and photothermal effects during the deactivation process. Therefore, the temperature increasing profiles under laser irradiation (808 nm, 1 W/cm^2^) in vitro was monitored to evaluate the photothermal efficiency of PPH@ICG. As shown in Figure 2a,b, the temperature of free ICG and PPH@ICG aqueous solution showed a quick temperature increase. The maximal temperature change of free ICG (80 μg/ml) and PPH@ICG (80 μg/ml) at 5 min could reach to 55.5 and 64.9°C, respectively. The photothermal efficiency of ICG encapsulated in NPs was enhanced slightly, which was consistent with previous reports that ICG‐containing micelles was more efficient to trigger laser‐dependent temperature increase than free ICG. The thermo‐sensitive micelles of the PPH@ICG (40 μg/ml) transformed from micellization to demicellization in the NIR laser‐triggered high‐temperature (up to 45.9°C) which was far above the LCST. This indicated that NIR laser stimulus was an effective tragedy to enhance drug release of PPH@ICG. In addition, the maximal temperature change of PBS only increased 1.7°C. As shown in Figure 2c, the thermal images were used to present the temperature variation of the PPH@ICG, which was consistent with the photothermal heating curves of PPH@ICG. As shown in Figure 2d, PPH@ICG showed the photothermal stability, which was favorable for PTT. The photo‐thermal conversion efficiency (*η*) of PPH@ICG was calculated to be 45.4%.

The standard curve of 5Fu is shown in Figure 2e. In our study, the drug EE and loading efficiency (EF) of 5Fu in PPH@5Fu@ICG were 68.3% and 19.6%, respectively. The standard curve of ICG is shown in Figure S5. Meanwhile, the drug EE and EF of ICG in PPH@5Fu@ICG was 53.1% and 10.3%, respectively. We further explored the NIR laser (808 nm, 1 W/cm^2^, 5 min) triggered 5Fu release. As shown in Figure 2f, PPH@5Fu@ICG treated with NIR show a significantly enhanced drug release. 5Fu released from PPH@5Fu@ICG was boosted at 15 min from 2.6% to 14.4% following 5 min NIR irradiation. NIR laser‐triggered 5Fu release was observed when the NIR laser was repeated at 7 h and 13 h, in which 5Fu release was increased from 23% to 33.6% and from 41.6% to 52.3%, respectively. Finally, 56.9% 5Fu was released after 24 h, which was about four times higher than PPH@5Fu@ICG without laser treatment. When the temperature is 43.4°C (higher than the temperature sensitive copolymer LCST), the original protective hydrophilic shell gradually precipitates from the aqueous solution due to phase transition, and then adsorbs on the hydrophobic core surface, resulting in the “core–shell” structure destruction of the micelle. In the process of phase transition, the water molecules in the micelle will also be squeezed out with the collapse of the hydrophilic shell. Meanwhile, some drug molecules will also be excreted, so that the release amount and release rate of thermo‐sensitive polymer drug‐loaded micelles at 45°C are significantly higher than those at 25°C.

### 3.2 In vitro cellular uptake

**FIGURE 3:**
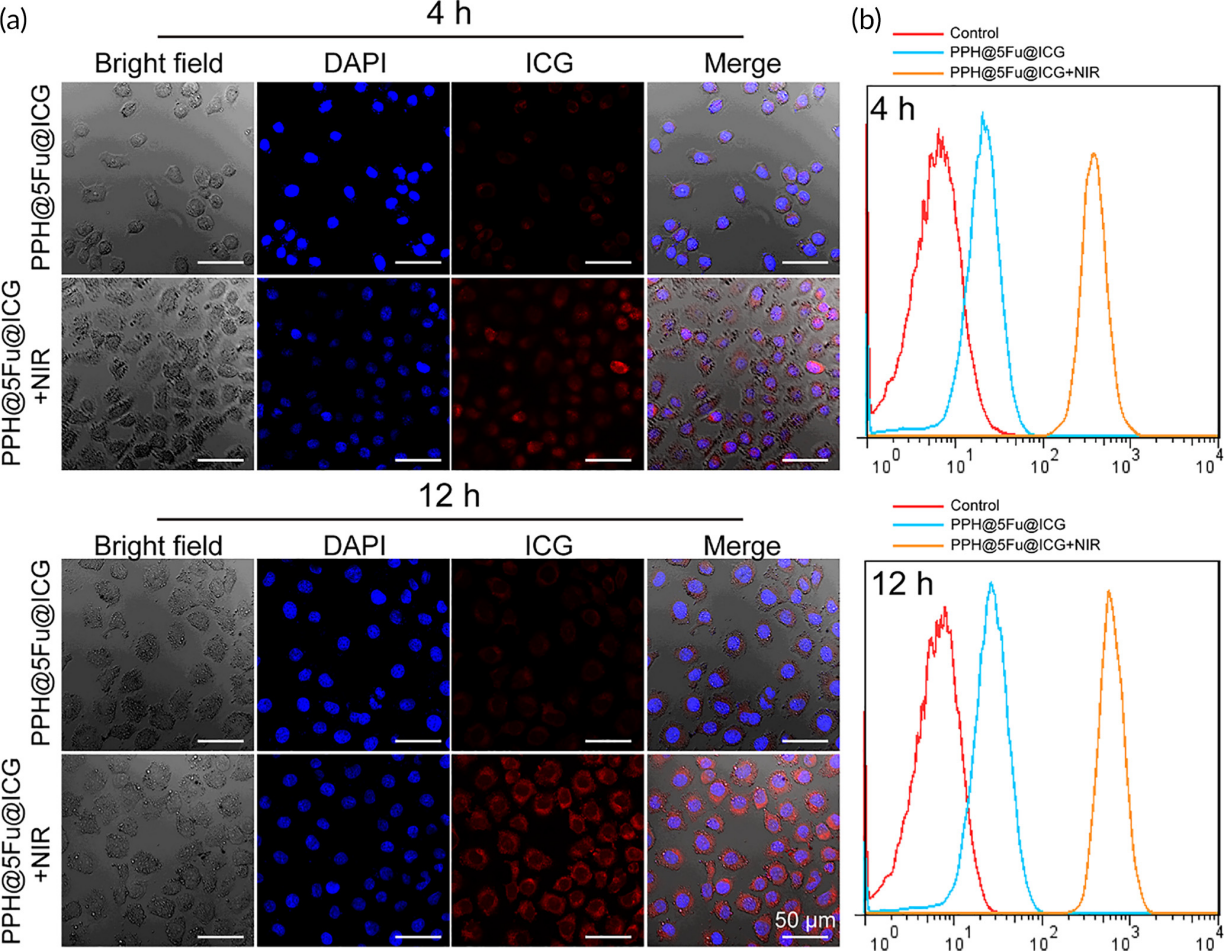
In vitro cellular uptake. (a) Confocal laser images of AGS cells after incubation with PPH@5Fu@ICG and PPH@5Fu@ICG + NIR for 4 and 12 h. (b) Endocytosis of PPH@5Fu@ICG and PPH@5Fu@ICG + NIR by AGS cells at 4 and 12 h post‐incubation via flow cytometry. Experiments were repeated three times. 5Fu, 5‐fluorouracil

Confocal laser scanning microscope was utilized to test the intracellular distribution of micelles and NIR laser‐triggered endosomal disruption in cells. As shown in Figure 3a, the fluorescent of DAPI and ICG were co‐localized without NIR irradiation, which implied that micelles were mainly localized in the cell cytoplasm. Meanwhile, the fluorescence of ICG and DAPI could be clearly detected in AGS cells after NIR laser irradiation (808 nm, 1 W/cm^2^, 5 min), which indicated that the NIR irradiation enhanced the ICG release from the PPH@5Fu@ICG. Notably, high uptake of ICG is clearly observed in PPH@5Fu@ICG + NIR group, showing the enhancement of antitumor efficiencies via hyperthermia. After NIR laser irradiation (808 nm, 1 W/cm^2^, 5 min), the blue fluorescent emanated by endo/lysosomes was conglomerated compared to the punctuate distribution without NIR laser irradiation, which was likely due to the endo/lysosomal disruption induced by the high‐temperature. After 4 and 12 h incubation, intracellular red ICG fluorescence signal of the AGS cells incubated with PPH@5Fu@ICG with NIR irradiation was stronger than that of the same cells without NIR irradiation. These results demonstrated a significantly higher rate of cellular uptake of PPH@5Fu@ICG with NIR irradiation than that without NIR in the AGS cells.

To further explore the cellular internalization process of ICG, flow cytometry analysis was performed. As shown in Figure 3b, ICG fluorescence signals of PPH@5Fu@ICG with NIR laser (808 nm, 1 W/cm^2^, 5 min) were largely internalized by AGS cells compared with that without NIR. Quantification of mean fluorescence intensity further revealed that the mean fluorescence intensity in AGS cells treated with NIR irradiation was significantly higher than that without NIR irradiation at 4 and 12 h (Figure S6).

### 3.3 In vitro antitumor effect by NIR laser‐driven 5Fu release

**FIGURE 4:**
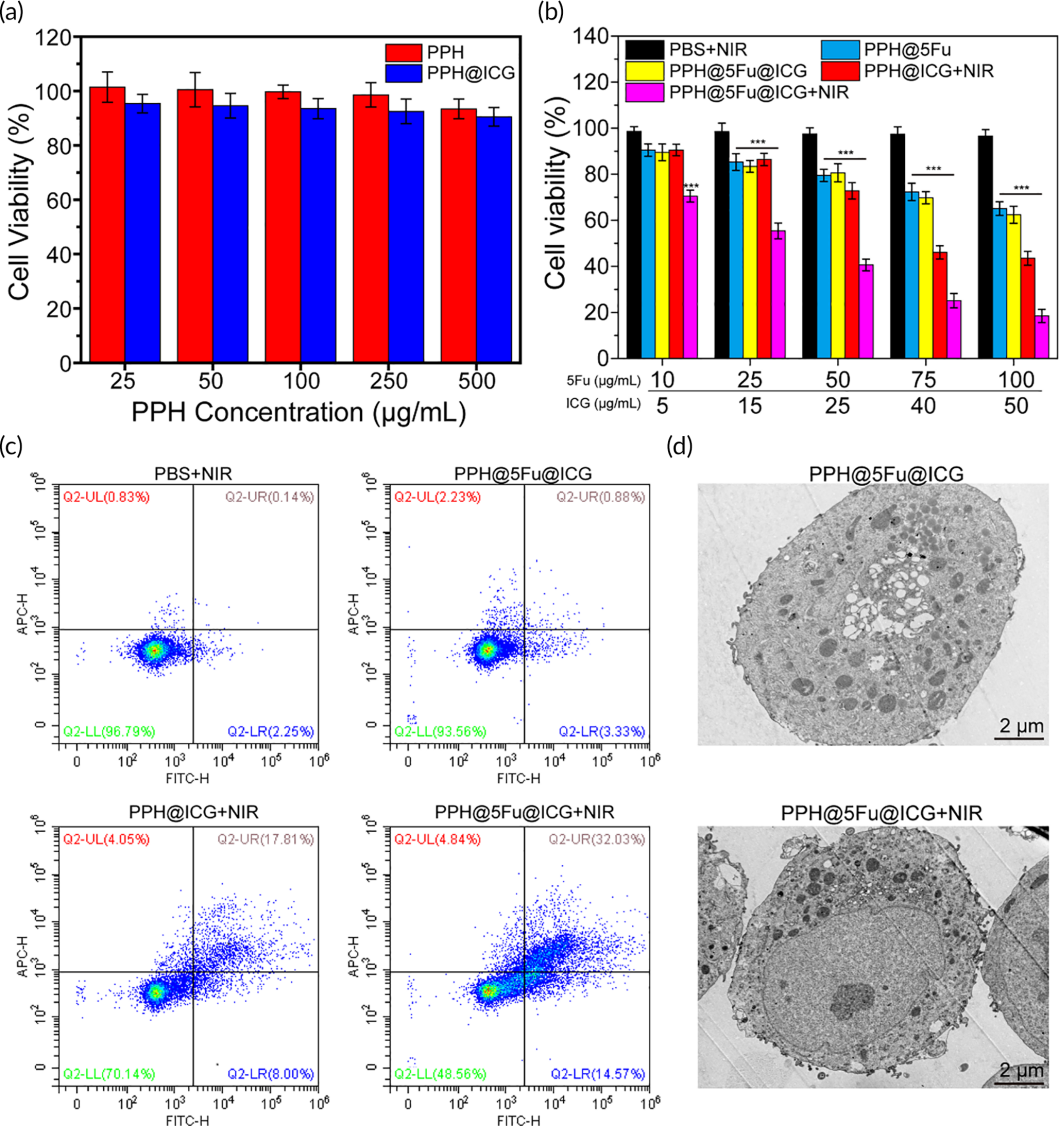
Enhanced cytotoxicity by NIR laser‐driven drug release. (a) Cell viability of AGS cells treated with PPH and PPH@ICG at various ICG concentrations. (b) Cell viability of AGS cells treated with PBS + NIR, PPH@5Fu, PPH@5Fu@ICG, PPH@ICG + NIR, and PPH@5Fu@ICG + NIR at various 5Fu or ICG concentrations. (c) Flow cytometry analysis of AGS cells after incubation with PBS + NIR, PPH@5Fu@ICG, PPH@ICG + NIR, and PPH@5Fu@ICG + NIR. Double stained cells were considered as late apoptotic/necrotic cells. (d) TEM images of AGS cells after incubation with PPH@5Fu@ICG and PPH@5Fu@ICG + NIR for 12 h. Experiments were repeated three times. 5Fu, 5‐fluorouracil; ICG, indocyanine green; NIR, near‐infrared

To directly investigate the biocompatibility of the nanoparticles, the viability of AGS cells after being exposed to PPH or PPH@ICG with various concentrations were evaluated. As shown in Figure 4a, the results showed that cell viability was greater than 90% and even the PPH concentration was increased to 500 μg/ml, indicating that PPH and PPH@ICG had no obvious toxicity in AGS cells. Next, the cell viability of PBS, PPH@5Fu, PPH@5Fu@ICG, PPH@ICG + NIR, and PPH@5Fu@ICG + NIR at various 5Fu or ICG concentrations to AGS cells was further investigated, respectively. As shown in Figure 4b, PPH@5Fu showed a little lower cytotoxicity than PBS. However, PPH@5Fu@ICG + NIR caused a better effect to kill AGS cells than PPH@5Fu@ICG under higher 5Fu concentration (75 μg/ml) and ICG concentration (40 μg/ml), which was likely due to the synergistic effects of abrupt drug release and chemo/PTT.^36^ NIR could induce the hyperthermia‐driven endosomal disruption (open) of PPH@5Fu@ICG under the relative high ICG concentration, resulting in the rapid release of 5Fu to kill AGS cells. However, PPH@5Fu@ICG under lower 5Fu concentration (10 μg/ml) and ICG concentration (5 μg/ml) could not induce sufficient hyperthermia to “open” the PPH@5Fu@ICG for rapid 5Fu release or directly kill AGS cells by heat.

The enhanced cytotoxic effects driven by NIR laser was further evaluated through cell apoptosis analysis. As shown in Figure 4c, the results showed that cell late apoptosis/necrosis was more efficiently induced by PPH@5Fu@ICG + NIR (32.0%) compared with cells treated with PPH@ICG + NIR (17.8%) and PBS (0.14%). The results evidently illustrated that NIR laser irradiation could obviously promote the effect of PPH@5Fu@ICG + NIR on apoptosis and cell death.^37^ As shown in Figure 4d, TEM results of tumor cell apoptosis displayed cell shrinkage, membrane blebbing, and chromatin condensation. Meanwhile, the PPH@5Fu@ICG nanoparticles with NIR irradiation group showed more black granular nanoparticles in AGS cells, which also implied much greater cellular uptake of the nanoparticle than without NIR irradiation.

### 3.4 In vivo fluorescence imaging and distribution

**FIGURE 5:**
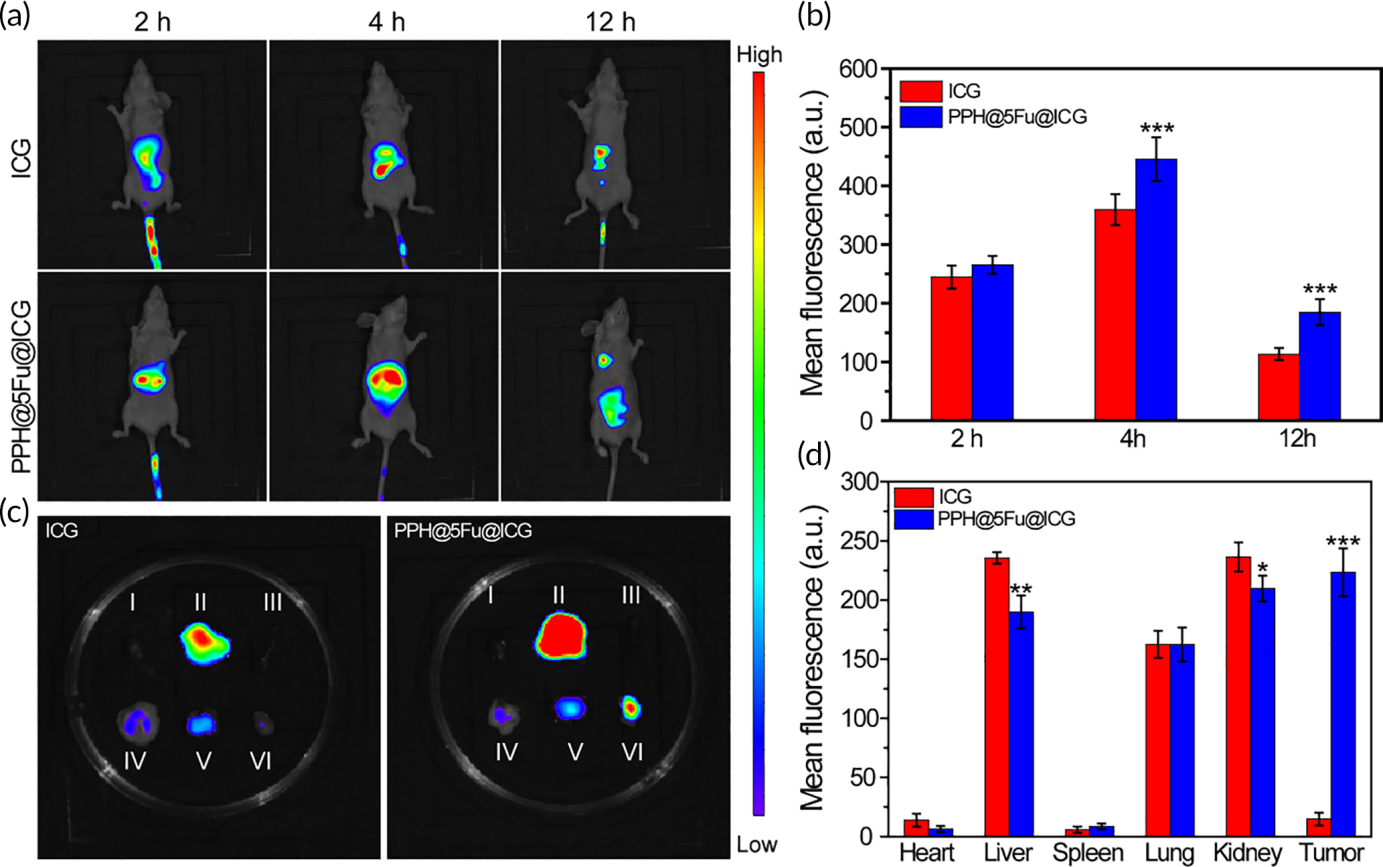
In vivo NIR fluorescence imaging AGS tumor‐bearing nude mice. (a) In vivo NIR fluorescence images of AGS tumor‐bearing nude mice 12 h receiving intravenous injection of ICG or PPH@5Fu@ICG. (b) Quantitation of the ICG fluorescence intensity of mice at different time points. (c) NIR fluorescence images of the isolated major organs and tumors 12 h post‐injection with ICG or PPH@5Fu@ICG (I, heart; II, liver; III, spleen; IV, kidney; V, Lung; VI, tumor). (d) Quantitation of ICG fluorescence intensity of individual organs and tumor. Experiments were repeated three times. 5Fu, 5‐fluorouracil; ICG, indocyanine green; NIR, near‐infrared

In vivo fluorescence imaging was utilized to investigate the real‐time distribution and intratumoral enrichment of PPH@5Fu@ICG micelles. In the gastric tumor‐bearing nude mice, the ICG fluorescence signals of PPH@5Fu@ICG group were much higher than pure ICG group at any time point (Figure 5a). ICG group was metabolized and cleared from blood circulation, and weak fluorescence was detected in tumor at 12 h only. In contrast, PPH@5Fu@ICG group was retained in vivo for a relatively long time. At indicated time points (2, 4, and 12 h), fluorescence signal intensity of tumors was evidently stronger in PPH@5Fu@ICG group than in ICG group (Figure 5b). PPH@5Fu@ICG micelles were largely accumulated in tumor tissue at 12 h due to the enhanced permeability and retention effect.^38^

Meanwhile, the biodistribution and tumor selectivity were also investigated via ex vivo imaging of dissected organs and tumors. Compared with ICG‐treated mice, enhanced fluorescent and PA signals could be detected in liver, kidney, and tumor of PPH@5Fu@ICG‐treated mice (Figure 5c). Collectively, PPH@5Fu@ICG has a long duration in the circulation and profound tumor‐specific accumulation with a peak at 4 h. As shown in Figure 5d, ICG group exhibited that free ICG mostly accumulated in liver and kidneys, and was barely detectable in the tumor. Interestingly, liver, lungs, and kidneys of ICG and PPH@5Fu@ICG showed a distinct accumulation of fluorescence signal, which was mainly related to metabolism of nanoparticles via these organs.^23^ However, PPH@5Fu@ICG group obviously increased the reservation of ICG and 5Fu in tumor. ICG FL signals could be apparently detected in tumor tissues, followed by kidneys and liver. Moreover, the averaged FL intensity of ICG at tumor site in PPH@5Fu@ICG group was about seven times higher than that in PPH@5Fu@ICG group. It was obvious from the results that PPH@5Fu@ICG could significantly improve the retention of ICG and 5Fu in the tumor through encapsulation of nanocarriers. Notably, ICG FL signals in the major organs of mice in ICG and PPH@5Fu@ICG groups were relatively weak because of relatively low dose of 5Fu accumulated in these organs except for the tumor.

The NIR laser‐mediated temperature increase in vivo was detected after direct injection of ICG and PPH@5Fu@ICG in tumors for 12 h. Figure S7A showed the infrared thermographic maps of mice after 5 min laser irradiation. As shown in Figure S7B, the tumor temperature of PPH@5Fu@ICG group rose to 43.9°C after 5 min laser irradiation, which could initiate drug release (~43°C) and cause an irreversible damage to the tumor tissues.^39^ However, the tumor temperature of ICG group can only reach to 38.3°C with same laser irradiation, which failed to cause irreversible damage to the tumor tissues.^40^

### 3.5 In vivo enhanced antitumor effect study

**FIGURE 6:**
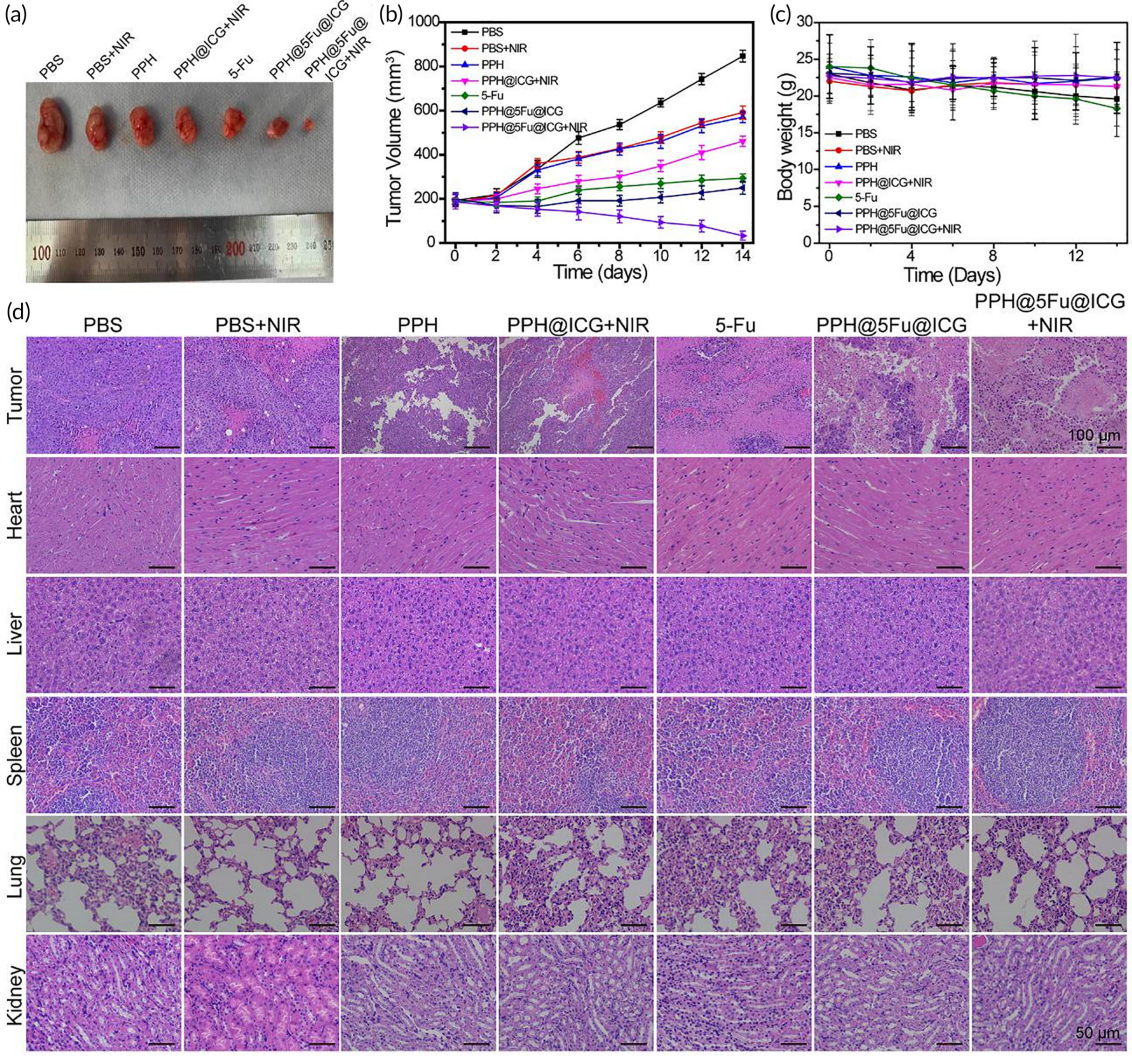
In vivo antitumor performance. (a) Photographs of representative tumors resected from different groups at 14 days. (b) Tumor growth curve in terms of volume. (c) Body weight of mice in different groups after treatments. (d) H&E staining images of tumor and major organs (heart, liver, spleen, lung, and kidney) slices after various treatments. Experiments were repeated three times

The antitumor effect of PPH@5Fu@ICG with 808 nm NIR irradiation in vivo was further evaluated in gastric tumor‐bearing nude mice. The mice treated with PBS + NIR or PPH showed the largest tumor size at day 14 compared with the other groups, which demonstrated that the growth of gastric tumors was not affected by laser irradiation or PPH (Figure 6a). The mice treated with PPH@ICG + NIR and 5Fu showed slow tumor growth with an averaged tumor size of 460.7 mm^3^ and 293.6 mm^3^ on day 14, respectively (Figure 6b). The detailed comparison was shown in Table S1. These results indicated that the low dosage of 5Fu and NIR irradiation have a positive effect on the inhibition of tumors by chemotherapy and PTT. Compared with other groups, the tumor size of PPH@5Fu@ICG + NIR group was the smallest, which achieved the best antitumor effect. These results indicated that PPH@5Fu@ICG + NIR group exhibited a significant improvement in antitumor efficacy due to the NIR‐triggered thermo‐chemotherapy synergy.^41^, ^42^

Moreover, the body weight of mice and H&E staining images of major organs were used to analyze treatment‐induced toxic side effects. As shown in Figure 6c, gastric tumor‐bearing nude mice treated with PPH@5Fu@ICG + NIR showed minimal weight loss than other groups, indicating that NIR‐induced thermo‐chemotherapy synergy could reduce toxic side effects for antitumor therapy through NIR triggered drug release and accumulation at tumor site and less 5Fu dosage on normal tissue. H&E staining images of major organs further indicated that all groups containing 5Fu were biocompatible and safe to nude mice (Figure 6d). In addition, H&E staining images of tumor indicated that PBS group showed the densest nuclear number of tumor cells. However, PPH@5Fu@ICG + NIR group showed obvious nuclei shrinkage and absent, and the tumor tissue was seriously damaged. Overall, the PPH@5Fu@ICG + NIR group achieved an optimal strategy for antitumor therapy without adverse effect or resistance through integrating real‐time tracing, controllable drug release, and chemo/PTT.

**FIGURE 7:**
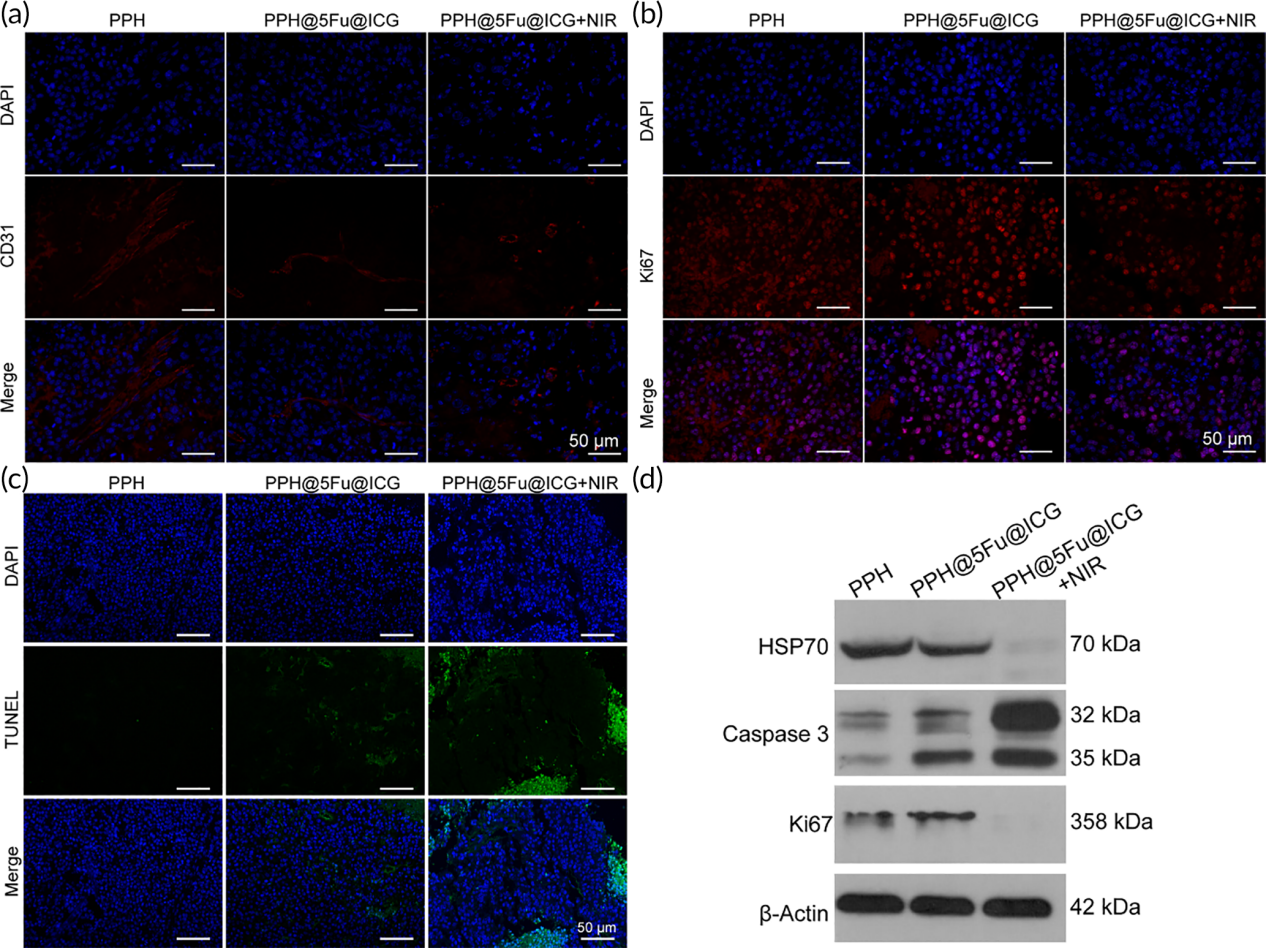
(a) Immunofluorescence of CD31 in the tumors. (b) Immunofluorescence of Ki67 in the tumors. (c) Immunofluorescence of TUNEL in the tumors. (d) WB analysis of HSP70, Caspase‐3, and Ki67. Experiments were repeated three times. HSP70, heat shock protein 70; WB, western blotting

The immunohistochemical staining of CD31 further supported the best antitumor effect of PPH@5Fu@ICG + NIR. CD31 was a platelet‐endothelial cell adhesion molecule with a molecular weight of 130 kDa, and was a transmembrane glycoprotein. CD31 was mainly used to evaluate tumor angiogenesis. As shown in Figure 7a, compared with the PPH group, all treatment groups could reduce the blood vessel density in tumor tissue with different degrees. Among them, PPH@5Fu@ICG + NIR showed a more significant inhibitory effect on angiogenesis. In addition, the proliferation of tumor cells was also investigated by Ki67 immunohistochemical staining (Figure 7b). The results showed that the expression of Ki67 in the PPH@5Fu@ICG + NIR group is lower than that in the other groups, indicating that the PPH@5Fu@ICG + NIR treatment group is the most effective in reducing tumor cell proliferation. All results were consistent with the previous results of tumor size, which further confirmed that PPH@5Fu@ICG + NIR had a good therapeutic effect in vivo from the histological level.

Treatment‐induced apoptosis by each group (PPH, PPH@5Fu@ICG, and PPH@5Fu@ICG + NIR) was analyzed using a TUNEL assay. As shown in Figure 7c, significant tumor cell apoptosis was observed in mice treated with PPH@5Fu@ICG and PPH@5Fu@ICG + NIR groups. PPH@5Fu@ICG + NIR group showed distinct superiority over PPH and PPH@5Fu@ICG groups. Western blotting (WB) of heat shock protein 70 (HSP70), Caspase‐3, and Ki67 was used to analyze the antitumor mechanisms of PPH@5Fu@ICG. HSP70 was an evolutionarily conserved chaperone protein under stress conditions, which played an important antiapoptotic role.^43^ As shown in Figure 7d, HSP70 expression of PPH@5Fu@ICG + NIR group was significantly inhibited compared to PPH and PPH@5Fu@ICG groups, which indicated that the mechanism of NIR‐induced photothermal chemotherapy is expression of HSP70. Caspase‐3 was a critical mediator of apoptosis, and enhanced caspase‐3 indicated higher degrees of tumor cell apoptosis.^44^ Ki67 could predict cancer progression as a marker of cell proliferation, and the high expression of Ki67 indicated more proliferating tumor cells.^45^ Meanwhile, PPH@5Fu@ICG + NIR group showed over‐expression of Caspase‐3 and low‐expression of Ki67 protein, which was due to the great inhibition of tumor growth and low side effect of NIR‐induced photothermal chemotherapy.^46^ The underlying mechanism of hyperthermia‐induced cell death may be that up‐regulate of HSP70 gene expression and inhibits caspase‐3 activation and down‐regulate of Ki67, resulting in mitochondrial instability yielding mitochondrial damage.^47^ In summary, PPH@5Fu@ICG nanoparticles achieved an excellent tumor ablation effect with a low dose of 5‐Fu and NIR irradiation in vivo, which could be traced by ICG NIR fluorescence imaging.

## 4 CONCLUSION

In summary, we successfully developed a novel thermo‐responsive 5‐fluorouracil/ICG‐coloaded micelles (PPH@5Fu@ICG) assembled by amphiphilic copolymers with excellent size distribution and high drug EF. Our results showed that PPH@5Fu@ICG exhibited excellent temperature response and NIR laser‐controlled drug release. We evaluated PPH@5Fu@ICG‐mediated chemo/photothermal therapeutic effect both in vitro and in vivo. The cell viability of integrated therapy mediated by the PPH@5Fu@ICG + NIR with 5Fu (75 μg/ml) and ICG (40 μg/ml) was the lowest among all of the groups (25.1 ± 3.1%). With external NIR laser stimulation, PPH@5Fu@ICG internalized in AGS cell‐induced local hyperthermia, endosomal disruption, rapid drug release, and caused enhanced effect to kill cancer cells. In vivo antitumor activities indicated PPH@5Fu@ICG could reach higher antitumor activity compared with free 5Fu and PPH@ICG for AGS tumor cells, and suppressed AGS tumor growth in vivo. Notably, the PPH@5Fu@ICG + NIR group could upregulate the expression of Caspase‐3, and downregulate the expression of Ki67 and HSP70. Moreover, the proposed approach for NIR laser‐induced nanomicelles with uniform nanoparticulate network may open new horizons for advanced and stimuli responsive therapeutic platforms.

## AUTHOR CONTRIBUTIONS

**Taicheng Zhou:** Conceptualization (equal); methodology (equal); writing – original draft (equal). **Lili Wu:** Conceptualization (equal); methodology (equal); writing – original draft (equal). **Ning Ma:** Conceptualization (equal); investigation (equal); methodology (equal). **Fuxin Tang:** Investigation (equal); methodology (equal); resources (equal). **Jialin Chen:** Formal analysis (equal); investigation (equal); methodology (equal). **Zhipeng Jiang:** Data curation (equal); formal analysis (equal); methodology (equal); software (equal). **Yingru Li:** Data curation (equal); methodology (equal); software (equal). **Tao Ma:** Formal analysis (equal); methodology (equal). **Na Yang:** Funding acquisition (equal); resources (equal); writing – review and editing (equal). **Zhen Zong:** Funding acquisition (equal); resources (equal); writing – review and editing (equal).

## CONFLICT OF INTEREST

There are no conflicts to declare.

### PEER REVIEW

The peer review history for this article is available at https://publons.com/publon/10.1002/btm2.10368.

## Supporting information


**Appendix S1** Supporting information

## Data Availability

Data available on request due to privacy/ethical restrictions.
