# Supplementary material for: Photothermally responsive theranostic nanocomposites for near‐infrared light triggered drug release and enhanced synergism of photothermo‐chemotherapy for gastric cancer
Source: Bioeng Transl Med. 2022 Jul 12;8(1):e10368. doi: 10.1002/btm2.10368 (PMC9842049; doi:10.1002/btm2.10368)
Supplement: Supplementary file 1 — Appendix S1 Supporting information [file BTM2-8-e10368-s001.docx]

**Supporting Information**

**Photothermally responsive theranostic nanocomposites for near-infrared light triggered drug release and enhanced synergism of photothermo-chemotherapy for gastric cancer**

Taicheng Zhou^a 1^, Lili Wu^b 1^, Ning Ma^a^, Fuxin Tang^a^, Jialin Chen^a^, Zhipeng Jiang^a^, Yingru Li^a^, Tao Ma^a^, Na Yang^c *^, Zhen Zong^d *^

^a^ Department of Gastroenterological Surgery and Hernia Center, the Sixth Affiliated Hospital of Sun Yat-sen University, Guangdong institute of Gastroenterology, Guangdong Provincial Key Laboratory of Colorectal and Pelvic Floor Diseases, Supported by National Key Clinical Discipline, Guangzhou, 510655, China.

^b^ Department of Medical Ultrasonics, Third Affiliated Hospital of Sun Yat-sen University, Guangdong Key Laboratory of Liver Disease Research, Guangzhou, 510630, China.

^c^ Department of Clinical Laboratory, Guangzhou First People's Hospital, School of Medicine, South China University of Technology, Guangzhou, 510180, China.

^d^ Department of Gastroenterological Surgery, the Second Affiliated Hospital of NanChang University, Jiangxi, 330006, China.

^1^ These two authors contributed equally to this work.

* Corresponding author: Na Yang, E-mail: fairyflora@126.com; Zhen Zong, E-mail: [zongzhenmd@126.com](mailto:zongzhenmd@126.com)

For TEM observation, 1 mL PPH and PPH@5Fu nanoparticles (1 mg/mL) was filtered by 0.45 μ m filter membrane. The filtrate was dripped on were dripped on to 200 mesh copper grids coated with carbon. After drying naturally in an air, the morphology of nanoparticles was investigated by high resolution transmission electron microscopy (TEM).

**Supporting experimental section**

**1.1 The photothermal conversion efficiency**

The photothermal conversion efficiency (η) of PPH@ICG can be calculated according to the following equation:

$$\text{η=}\frac{\text{hA(}{\Delta T}_{\text{max, mix}}\text{-}\text{ }{\Delta T}_{\text{max, H2O}}\text{)}}{\text{I(1-}{10}^{-A\lambda}\text{)}}$$

where h is the heat transfer coefﬁcient, A is the surface area of the container, ΔT_max, mix_ and ΔT_max, H2O_ are the temperature change of the PPH@ICG dispersion and solvent (water) at the maximum steady-state temperature, respectively, I is the laser power, and A_λ_ is the absorbance of PPH@ICG at 808 nm.

**Table S1**. Tumor growth volume of different groups

| Tumor Volume (mm^3^) | | | | | | | |
| --- | --- | --- | --- | --- | --- | --- | --- |
|  | PBS | PBS+NIR | PPH | PPH@ICG  +NIR | 5Fu | PPH@5Fu  @ICG | PPH@5Fu  @ICG+NIR |
| 0 day | 192.8 | 183.2 | 183.5 | 186.5 | 189.5 | 199.7 | 186.7 |
| 2 day | 219.4 | 215.4 | 205.5 | 199.5 | 184.2 | 169.9 | 167.7 |
| 4 day | 335.6 | 360.2 | 330.2 | 245.5 | 190.2 | 165.6 | 152.8 |
| 6 day | 475.8 | 387.9 | 381.4 | 280.1 | 239.5 | 191.6 | 141.5 |
| 8 day | 536.4 | 428.8 | 425.6 | 301.1 | 255.4 | 191.4 | 119.9 |
| 10 day | 635.9 | 478.6 | 459.9 | 348.8 | 269.9 | 206.8 | 93.6 |
| 12 day | 742.5 | 545.1 | 530.4 | 409.8 | 284.5 | 227.7 | 76.6 |
| 14 day | 846.7 | 590.9 | 569.9 | 460.7 | 293.6 | 249.8 | 32.8 |


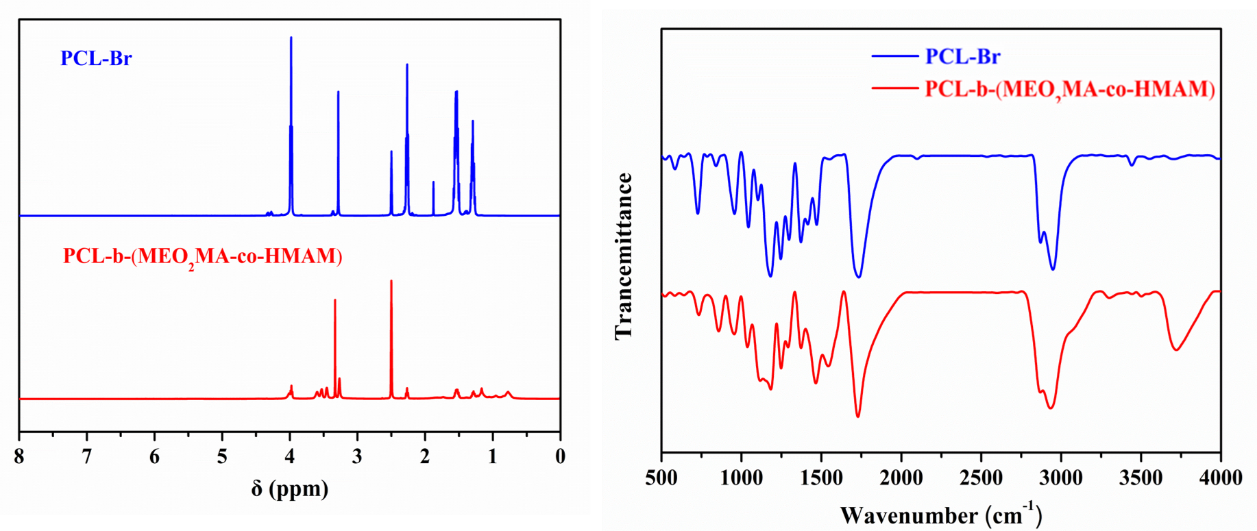


**Figure S1.** (A) ^1^H NMR characterization of the PCL-Br and PCL-*b*-(MEO_2_MA-co-HMAM). (B) FTIR spectrometry of the PCL-Br and PCL-*b*-(MEO_2_MA-co-HMAM).

**
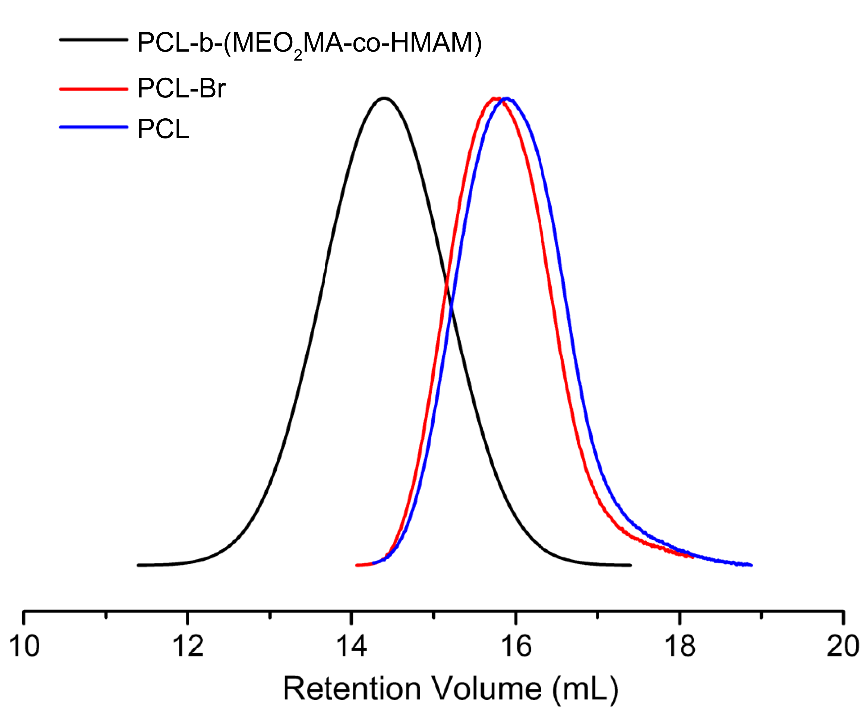
**

**Figure S2.** GPC curve of PCL, PCL-Br and PCL-*b*-(MEO_2_MA-co-HMAM).

**
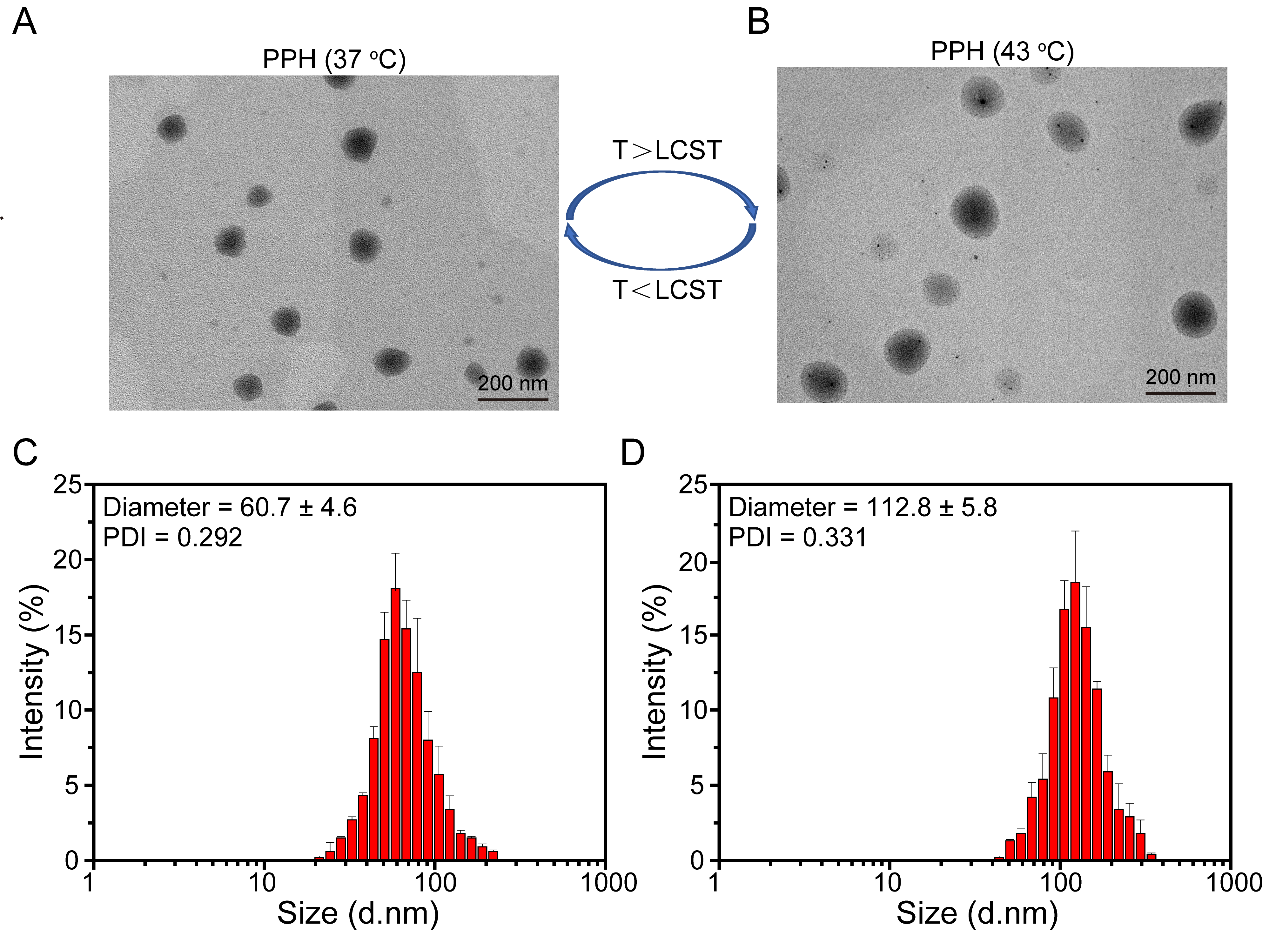
**

**Figure S3.** Characterization of the temperature-sensitive PPH nanoparticles. (A) TEM images of PPH nanoparticles at 37^o^C. (B) TEM images of PPH nanoparticles at 43^o^C. (B) Size distribution of PPH nanoparticles at 37^o^C. (D) Size distribution of PPH nanoparticles at 43^o^C.





Figure S4. DSC curves of PPH@5Fu@ICG nanoparticles.





**Figure S5.** The standard curve of ICG.

**

**

**Figure S6.** Quantification of mean fluorescence intensity.

**
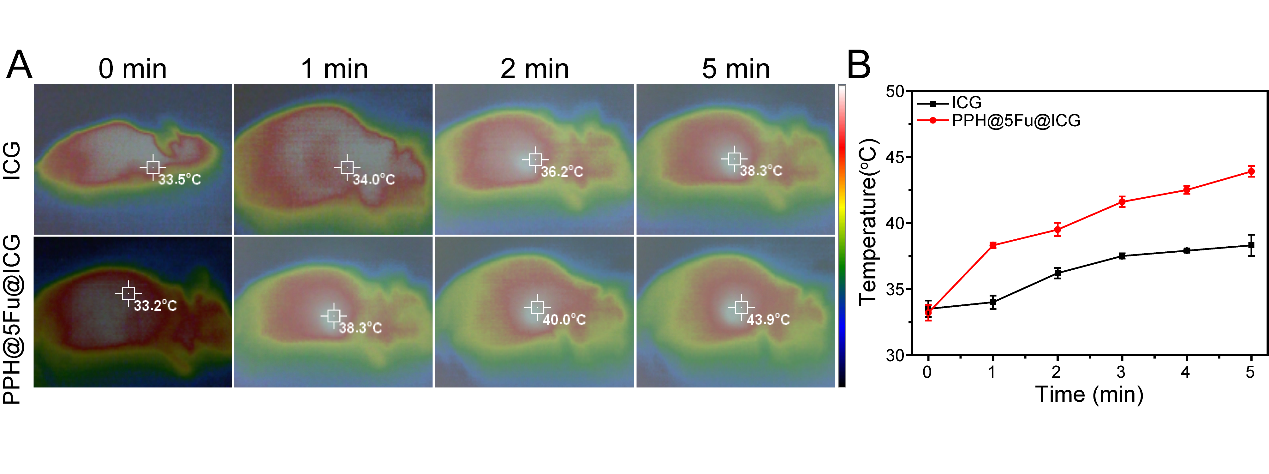
**

**Figure S7.** (A) Infrared thermo-graphic maps of mice after 5 min irradiation. (B) Temperature increasing profiles of laser-irradiated (808 nm, 1 W/cm^2^, 5 min) tumour tissues 12 h after injection of ICG or PPH@5Fu@ICG.
